# Can circularity support net-zero agriculture: an exploratory case

**DOI:** 10.1093/af/vfaf019

**Published:** 2025-09-19

**Authors:** Tim McAllister, Ben Ellert, Henry Janzen

**Affiliations:** Agriculture and Agri-Food Canada, Lethbridge Research and Development Centre, Lethbridge, AB, CanadaT1J 4B1; Agriculture and Agri-Food Canada, Lethbridge Research and Development Centre, Lethbridge, AB, CanadaT1J 4B1; Agriculture and Agri-Food Canada, Lethbridge Research and Development Centre, Lethbridge, AB, CanadaT1J 4B1

**Keywords:** agriculture, circularity, climate change, greenhouse gas emissions

**Implications**

- A durable net-zero Canadian agriculture by 2050 is a formidable challenge, even if circularity principles are employed.
- A net-zero target gives impetus to restructure agricultural land use and livestock systems for multiple societal aims.
- A target of net-zero by 2050 demands scientific tools and infrastructure to verify progress.

## Introduction

Emissions of greenhouse gases (GHGs), notably CO_2_ from fossil fuel combustion, are increasingly altering the radiative balance of Earth’s atmosphere, leading to dangerous shifts in global climate (Ripple et al., 2024). In response to this human-inflicted threat, Canada, like other countries, has committed to drastically curtailing its agricultural emissions, having set a target of net-zero emissions by 2050 (Government of Canada, 2024). Currently, agriculture accounts for about 10% of Canada’s annual GHG emissions (Environment and Climate Change Canada, 2024a). Globally, Canada ranks among the 10 nations with the highest agricultural GHG emissions (Sutton et al., 2024). As a result, agricultural sectors in Canada are developing strategies in an effort to reach net-zero emission targets by 2050 (e.g., Dairy Farmers of Canada; Canadian Alliance for Net-Zero Agri-food).

Farming has several distinctive features that distinguish it from other societal ventures in the pursuit of net-zero emissions. Firstly, unlike other sectors, livestock production is a prominent source of CH_4_ and N_2_O, not just CO_2,_ as in other sectors (Khalil et al., 2024). These gases emanate largely from microbial processes, which direct carbon and nitrogen flows through farm ecosystems. Secondly, farmlands also withdraw GHGs, notably by storing CO_2_-derived C in augmented soil and vegetation. These enhanced carbon “sinks” can both advance and hinder the quest for net-zero; as the climate warms, portions of the carbon reserves may be released into the atmosphere through decay or by fire, contributing to climate change (Dooley, 2024). Thirdly, farms produce food, including livestock products, that are indispensable to human welfare and equity (Haring et al., 2023). It has been proposed that GHGs emissions from agricultural lands could be eliminated if they were returned to “Nature” (Monbiot, 2022), but adoption of such measures may be limited as food security will almost certainly take precedence over climate mitigation. Carbon that is stored through strategies that detract from food production will have negative socio-economic implications, and stored carbon will only be retained if these areas remain undisturbed indefinitely. Policy incentives that pay for ecosystem services or rewilding programs are only like to be viable if they do not threaten food security or the sustainability of agricultural systems. Consequently, the introduction of circularity principles in integrated livestock—crop systems is likely to make the greatest contribution towards achieving net-zero objectives.

## The Target: What is Net-Zero?

Definitions and ways of calculating net-zero abound (Christiansen et al., 2023; Green and Reyes, 2023; Rogelj, 2023), but implicit in most definitions is the assumption that the global atmosphere is the final arbiter. For a sector to achieve net-zero status, its net emission of radiative-forcing gases to the global atmosphere must be less than or equal to zero.

Under these conditions, metric-weighted anthropogenic greenhouse gas (GHG) emissions are balanced by metric-weighted anthropogenic GHG removals over a specified period. (where anthropogenic = derived from human activity).

Although arithmetically simple, estimating net zero in agricultural systems is challenging. For our purposes here, we make the following simplifying assumptions:

- Emissions and removals include all processes arising from human activity on lands within the “total farm area” by Statistics Canada (2024). In 2021, this land covered some 62 million ha, of which about 38 million ha was cropland, with large areas also in pastures and interspersed wood- and wet-lands.
- All exchanges of CH_4_, N_2_O, and CO_2_ occurring on those lands are included, with the combined global warming potential of these gases expressed as CO_2_ equivalents (CO_2_e).
- Emissions of CH_4_ and N_2_O are assumed to have global warming potentials of 27 and 273 CO_2_e, respectively, corresponding to a 100-year time frame (IPCC, 2022), acknowledging the merits of alternative values corresponding to shorter time frames.
- For estimation of emissions and withdrawal, the relevant boundaries are set to include all exchanges from the manufacture of inputs through processes on farms and ending with, but not including, the fate of the products.
- As argued by Allen et al. (2022), net-zero must be “durable”—it must persist for at least several decades—so we arbitrarily set t = 30 years (2050–2080).

## Where are We Now?

**Figure 1.**
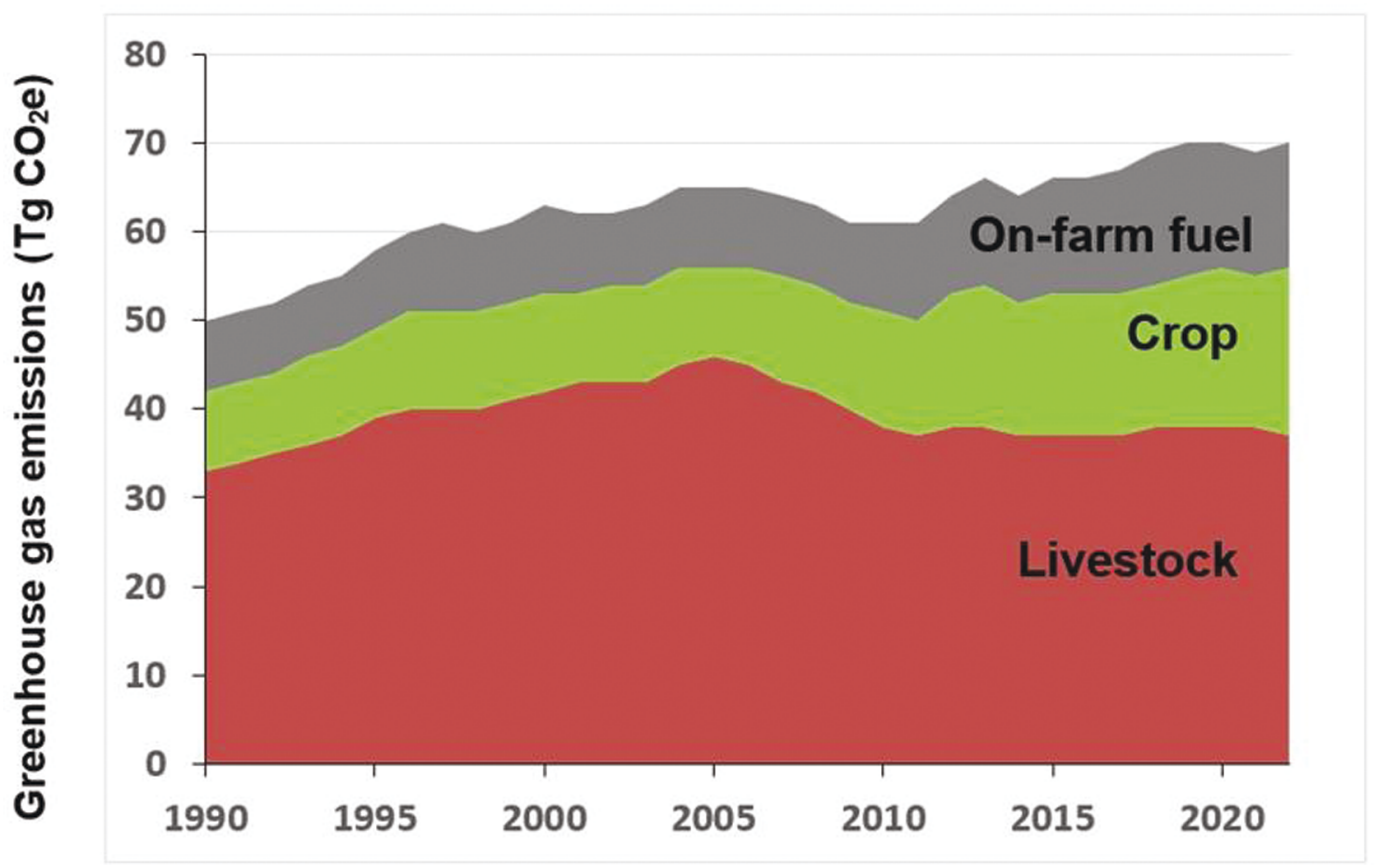
Greenhouse gas emissions from Canadian agriculture, 1990–2022. Environment and Climate Change Canada (2024b) National Inventory Report 1990–2022: Greenhouse Gas Sources and Sinks in Canada (www.canada.ca/en/environment-climate-change/services/climate-change/greenhouse-gas-emissions/inventory.html).

To assess the prospects of achieving net-zero, we first need to take stock of the current status with respect to emissions and removals. According to estimates in Environment and Climate Change Canada (2024a), emissions of GHG from agriculture in Canada amounted to about 70 Tg CO_2_e in 2022, of which about half originated from animal agriculture, and the remainder, in roughly equal amounts, from crop production and on-farm fuel use (Figure 1; Environment and Climate Change Canada, 2024b). These values are likely underestimates as they may not fully capture CO_2_ emissions from energy used to transport and manufacture inputs, nor that used to send products to market (Desjardins et al., 2020). Estimates projected for agriculture in 2030 are only 1% less than those in 2005 (Environment and Climate Change Canada, 2022), demonstrating the difficulty of drastically curtailing emissions while increasing agricultural production.

**Figure 2.**
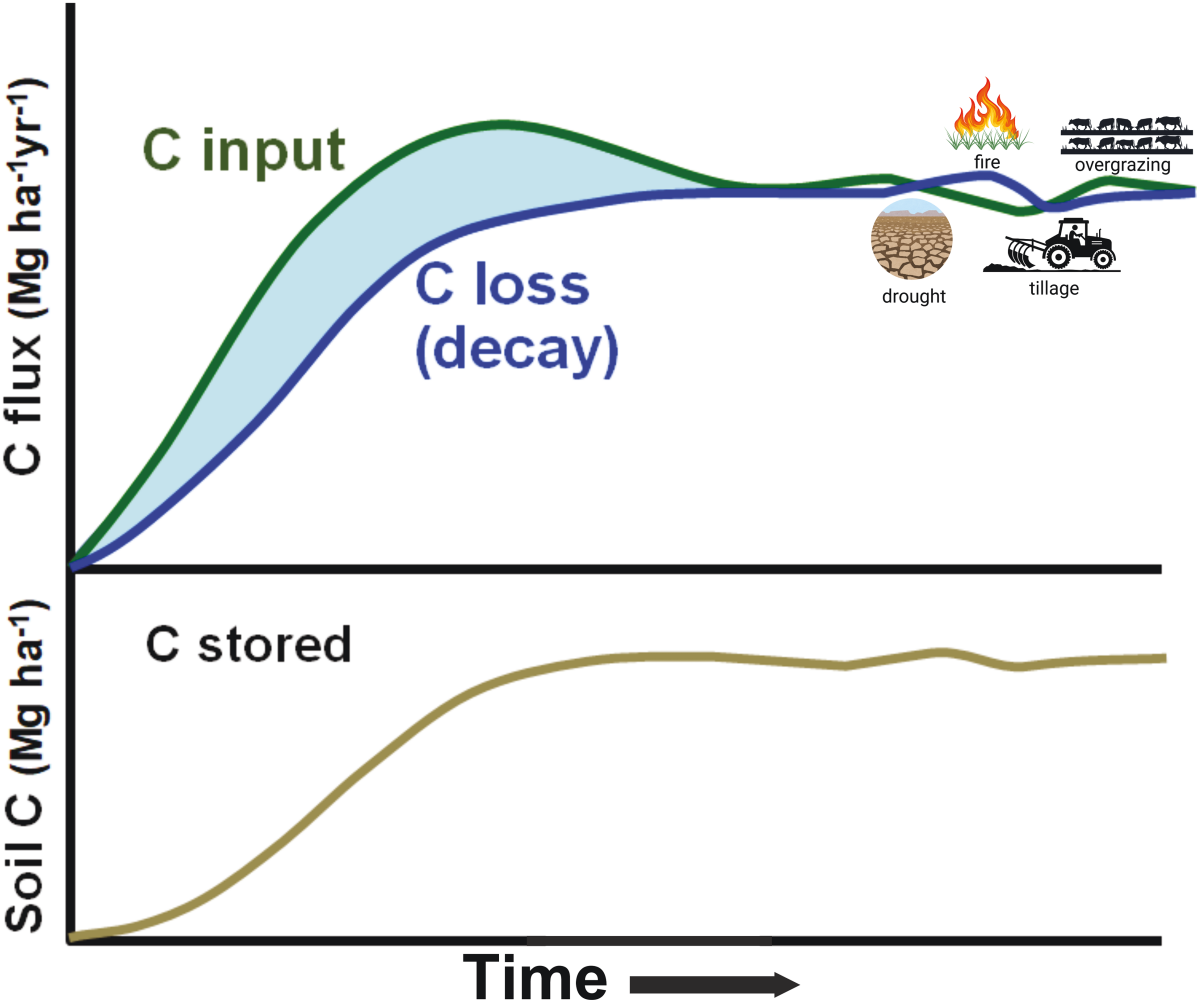
Conceptual diagram showing finite responses in soil organic C to improved management (based on Odum, 1969). The temporal pattern of the response and its magnitude will vary among environments, depending on factors such as climate, soil type, and prior management practices. Ecosystem C stocks rarely reach a true steady state, but rather fluctuate continually near an equilibrium, reflecting responses to changes in climate, management, vegetation, and other factors.

Canadian agriculture, however, can conceivably also remove GHGs from the atmosphere, notably by improving management practices to augment soil carbon (Environment and Climate Change Canada, 2022). According to the National Inventory, based on model outputs, removals of atmospheric CO_2_ by cropland soils have amounted to about 20 Tg CO_2_e in recent decades, though values fluctuate markedly from year to year, ranging from removal (i.e., sink on land) of 45 Tg CO_2_e in 2014 to an addition (i.e., net loss) of 18 Tg CO_2_e in 2022. These fluctuations reflect changes in land use and management, as well as episodic weather events such as drought, on soil carbon stocks (Figure 2) . Measuring small annual changes (typically about 20 Tg CO_2_e) in a vast soil carbon pool (likely more than 10,000 Tg CO_2_e) is inherently challenging. The variability of year-to-year estimates also emphasizes the reversibility of soil C sinks and their potential vulnerability to future climate change. Additional losses of C (about 1.7 Tg CO_2_e in 2022) occur from the ongoing conversion of forestland or grassland to cropland (Environment and Climate Change Canada, 2024b). Not yet included in estimates of soil C sinks are potential accumulations of inorganic C (Box 1). Additional sinks of C may occur in wetlands and afforested or reforested lands associated with agriculture. As C flows are circular, farmlands can be both sinks and sources, sometimes intermittently.

Box 1. **Atmospheric CO_2_ Removal Through Enhanced Rock Weathering**

Enhanced rock weathering (ERW) involves mining a silicate feedstock and then applying it to agricultural land as a dust, finely ground to maximize surface area, thereby promoting weathering and subsequent carbonate formation. Aside from removing atmospheric CO_2_, this practice may also help increase pH and release plant nutrients, but it also carries the risk of environmental contamination (Battersby, 2024). Increasingly, ERW is considered an alternative or supplement to other C-withdrawing practices, such as soil organic C sequestration, afforestation, and biochar application ( Table 1; Chiquier et al., 2025). Unlike other practices, notably organic C sequestration, ERW has no immediate limit on the amounts of carbonate that can be stored, at least in the coming decades. Despite its theoretical promise, however, ERW has not yet been adequately studied to confidently endorse its merits and estimate its capacity for atmospheric CO_2_ removal.

**Table 1.** Comparison of sequestration as soil organic carbon and as carbonate for reducing atmospheric CO_2_

	*Soil organic C sequestration*	*Sequestration as carbonate by ERW*
***Feasibility***	**proven**	**Unknown**
***Technological readiness level***	**High**	**Very low**
***Applicable cropland area in Canada***	**20 MHa**	**40 MHa**
***Likelihood of co-benefits***	***	^*^
***Likelihood of adverse environmental effects***	**Very low**	**Medium, depending on feedstock**
***Likelihood of crop productivity drag***	**Medium**	**Low**
***Permanence***	**impermanent**	**Permanent**
***Capacity for CO***_***2***_ ***removal, 25 years***	Small (SOC^*^ pool equilibration)	**Potentially much larger compared to SOC?**
***Expense, relative to business-as-usual***	**Low to medium; very low relative to ERW**	Medium to high; very low relative to DAC^*^

^*^ **SOC, soil organic carbon; DAC, direct air capture**.

Using these values, the current net emission of GHGs from agriculture in Canada may be roughly 50 Tg CO_2_e yr^-1^. Despite intensive research and policy initiatives, this net value seems not to have declined much, if at all, in recent decades. Consequently, to achieve a durable net-zero, Canadian farming systems must reduce emissions and increase removals by at least 50 Tg CO_2_e yr^-1^ from 2050 onwards.

## Our Thesis

**Figure 3.**
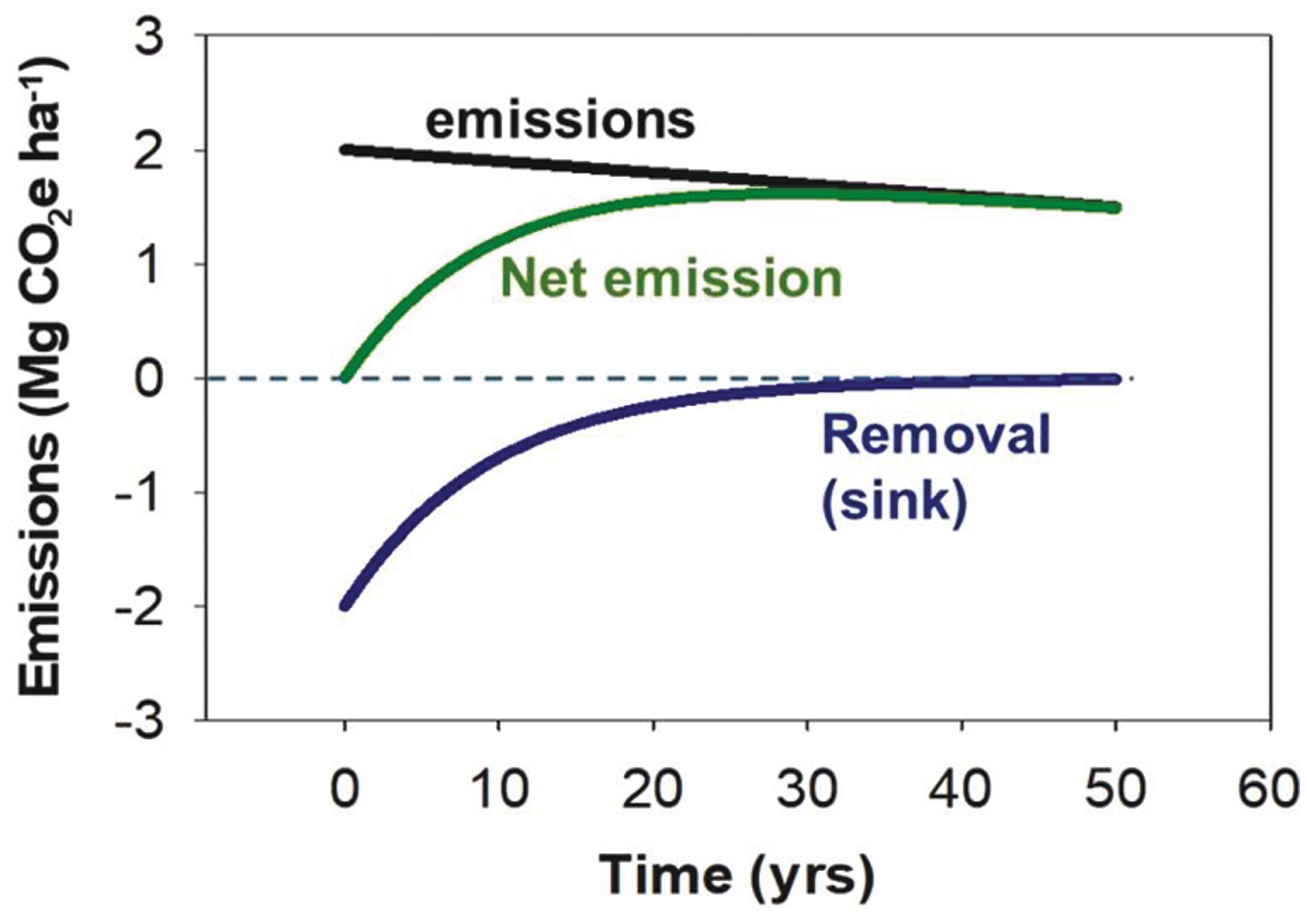
Conceptual illustration of the net benefits to atmospheric C from adoption of an improved, soil C-conserving practice, assuming continuing emissions of greenhouse gases, though at a gradually declining rate. In early phases after adoption, a C-conserving practice could ostensibly offset emissions, creating a net-zero status. But with time, as the soil approaches a new equilibrium, net emissions will increase again, even with declining total emissions.

Based on the preceding rationale, we contend that reaching a durable net-zero for Canada’s farming is a formidable challenge. We see no way of achieving this target while still furnishing essential products from our farmlands and maintaining rural communities. In short, it seems inconceivable that all emissions from agriculture can be squelched, so substantive “residual” emissions will remain and will need to be offset by equivalent terrestrial C withdrawals. Such withdrawals are finite and subject to reversal; emissions will continue, but withdrawals will inevitably decline (Figure 3). A durable net-zero, therefore, becomes an extravagantly optimistic goal that can likely only be achieved through transformative changes. Some transformative changes are likely to be broad in scope, while others will be regionally or even farm-specific. As a consequence, broad mitigation predictions will invariably be accompanied by a high level of uncertainty. As a result, transformative change practices must be evaluated on a case-by-case basis.

## Examples of Transformative Change

### Return large areas of arable land back to nature

Restoring some semblance of the pre-cultivation vegetation—grasslands, forests, or wetlands—can sharply curtail emissions of GHGs, while at the same time fostering C sinks, at least for a time. In effect, this amounts to a reversal of the changes enacted by the original commandeering of natural lands for agriculture. Obvious candidates for such a reversal are the vast areas of croplands deemed “marginal” for cropping, perhaps amounting to as much as 9 million ha in Canada (Liu et al., 2017). But there may also be ways of integrating such re-naturalized areas into productive, intensively managed croplands. For example, some researchers have proposed planting prairie strips among cropland fields (Kremen and Merenlender, 2018). Such restorative approaches, because of fragmentation, will not fully restore lands to pre-cultivation status, but may still allow for enhanced soil C storage. In some places, it may be possible to restore previously drained wetlands, leading to re-accumulation of soil C, reduced N_2_O emissions, but possibly elevated emissions of CH_4_ (Nyberg et al., 2022).

Always present, however, is the risk of “leakage”; restoring native vegetation inevitably sacrifices agricultural yield, often diverting production elsewhere through intensified farming (elevated emissions) or land use change (loss of stored C). Achieving “net-zero” in one place, therefore, may come at the expense of enhanced emissions in another place, so the atmosphere sees little benefit. The issue of leakage is crucial, especially in light of growing demands for food worldwide; recent estimates suggest an increase of 10% to 62% will be required from 2010 to 2050 (Beans, 2022).

Another critical facet here is the question of permanence (Ogle et al., 2023; Zickfeld et al., 2023). There is little benefit in transforming current croplands into natural vegetation unless that restoration endures indefinitely, with little risk of its return to cropping. As with all such proposed practices, this inevitably engages economic dimensions and considerations.

### Develop multi-functional landscapes

**Figure 4.**
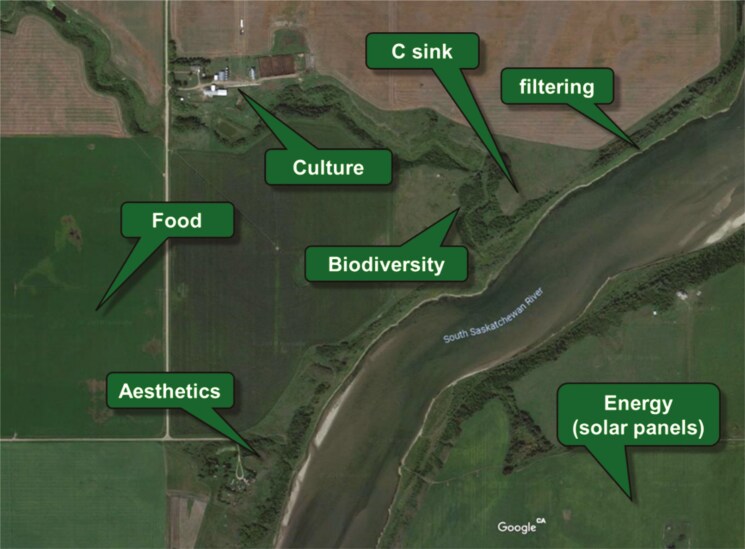
A hypothetical example of a multi-functional landscape, in which different locations are focused on diverse land functions, optimized for varying micro-climate, soil properties, and geographical features.

A second potential strategy, related to the first, is the deliberate re-arrangement of farming practices to create multi-functional landscapes. Here, different places within the landscape would be optimized and managed to support different socio-ecological functions, depending on local conditions. For example, some places might be designated primarily for intensively producing food, others to serve as C sinks, still others to promote biodiversity, remove pollutants from water, or serve other ecological functions (Figure 4). In some cases, it may even be possible to generate electricity through strategically placed wind turbines, solar arrays, biodigestors, or hydro generators. Agrivoltaics is an example of where various crops and livestock including bees, poultry, sheep, swine and cattle are produced on land under solar arrays, as in commercial and pilot projects underway in Europe, Asia, Africa, the America’s and Australia (Soto-Gomez, 2024), In this way, the landscape, as a whole serves manifold functions, while heeding advantages of localized topography, soil type, and microclimate to foster individual functions. For example, the best places for storing additional C may be, not be in the crop fields that are the subject of so many “sequestration” goals, but in associated wetlands, tree stands, or riparian areas (Taylor et al., 2019).

This strategy, however, demands a wholesale re-orientation of agricultural research and practice (Petit and Landis, 2023). As with any substantive transformation, re-aligning farming systems to promote multi-functional landscapes confronts imposing challenges, including the inevitable costs of transition, long-term maintenance, and their compatibility with large-scale farming practices.

### Perennialize cereal, pulse, and oilseed crops

A third potential contributor toward net-zero is to revert to using perennial rather than annual vegetative species (Entz and van Die, 2024). Perennials tend to store more carbon as they usually have a longer duration of photosynthesis and accumulate more below-ground biomass (Kuzyakov and Domanski, 2000). Historically, farming has typically converted perennial plant populations to annual species, often resulting in ecosystem C losses (Janzen, 2001) and reduced circularity. The primary obstacle to this transformation is that annual species, notably cereals and some oilseeds, remain our primary sources of food and other essential commodities. Efforts to develop perennial grain crops such as wheat, rye, triticale, oats, rice, and corn have been underway for decades (Sprunger et al., 2024), but agronomically viable alternatives that match the productivity of annual species remain years or decades away (Chapman et al., 2022). In the meantime, practices that mimic perennial species, such as cover cropping (where climatic constraints allow) and multi-cropping (Entz and van Die, 2024), may contribute to net-zero objectives.

### Re-think the place of animals on farms

Livestock, notably ruminants, have several relevant advantages as symbiotic biota in robust ecosystems, as they can provide economic returns and food from grasslands, thus conserving soil C and biodiversity. With appropriate grazing regimes, they mimic conditions under which grasslands evolved, thereby enhancing ecological viability and functionality (Cao et al., 2024) through enhanced circularity. Ruminants also promote the use of perennial forages in rotation with annual crops, conserving and augmenting soil C, but also decrease the need for synthetic fertilizer N through biological N fixation. Finally, if properly managed, their manure promotes the effective recycling of nutrients (Prairie et al., 2023), thereby reducing the need for energy-intensive synthetic fertilizers. Livestock also offer cyclical synergies with growing food on croplands, by productively recycling oil meals, hulls, crop residues, and other byproducts of food processing, thereby creating circular flows of energy and materials (Ominski et al., 2021; Franzluebbers and Hendrickson, 2024).

At the same time, however, imprudent reliance on livestock may also hamper progress toward net-zero as ruminants produce copious amounts of CH_4_ (Beauchemin et al., 2024). Their wastes, whether managed mechanically or voided directly on land, can be a significant source of N_2_O and CH_4._ Animal-based farming also typically requires more land and water per unit of food produced than plant-based farming. For these reasons, replacing animal-derived foods with plant-based equivalents has been widely advocated as a pathway to net-zero (Li et al., 2024), but such wholesale approaches are overly simplistic and overlook potential ecological costs.

For these reasons, the place of livestock in future farming systems is among the most controversial, complex questions, warranting intensified scrutiny and creativity (Van Oort et al., 2024; Whitmee et al., 2024). If there is to be substantive progress toward net-zero agriculture, the farming community—producers, researchers, educators, and agri-industry—will need to ponder unprecedented improvisation in establishing new relationships between land and livestock, with an emphasis on promoting the contribution of livestock to circularity.

### Identify and suppress N losses

Despite decades of research, the N balance of farmlands remains obscure. In Canada, as much as 40% of N entering Canadian farming systems is unaccounted, a value that has improved only marginally in recent decades (Karimi et al., 2020). One of the “leaks” in the N cycle is N_2_O. Although accounting for only a small fraction of N losses, N_2_O emissions are a symptom of inefficient N use on farms. Until we understand precisely where, how, and when N leaks from our systems, progress in reducing N inputs and N_2_O emissions will remain slow. Considering that N_2_O accounts for about a third of current agricultural emissions in Canada, progress toward net-zero targets will be unachievable without elucidating mechanisms of N loss through better measurements and practices. Foci of fruitful pursuits in research and farming may include the microbiology of nitrification and denitrification and the development and exploitation of N-fixing species (Box 2).

Box 2. **Potential Merits of Biological N_2_-Fixing Cereals for Reducing GHG Emissions**

Agriculture now depends heavily on industrially fixed nitrogen (N) to sustain productivity, partially replacing N removed in plant harvest. In Canada, farmers applied about 2.7 Tg N to land in 2022, a 2.6-fold increase since 1982 (FAOSTAT, 2025). These inputs are a prominent source of N_2_O from agricultural soils; moreover, the manufacture of the fertilizer is energy-intensive, generating CO_2_ and CH_4_ from fossil fuel used in its production. For these and other reasons, surplus N has been deemed to be among the most extreme violations of planetary boundaries (Steffen et al., 2015).

One way of reducing industrially fixed N use, and its adverse environmental consequences, while still satisfying crop N demands, is to rely more extensively on biologically fixed N. Symbiotic processes already supply most of the N required by legume crops: such as alfalfa and clover, and annual crops such as pea, lentil, and soybean (Karimi et al., 2020). These, however, represent a small proportion of crops used directly for human food. The extent to which cereal and non-legume oilseed crops can be induced to fix N has been a prominent research question for more than a century, with occasionally dubious results. Recently developed genetic techniques yield fresh opportunities for extending N-fixing capacity to prominent non-legume food crops, using various biological mechanisms (Pankievicz et al., 2019; Guo et al., 2023). Development of biological N-fixing cereals and other annual crops, therefore, shows some promise for reducing GHG emissions, but net benefit remains uncertain, given the complexity of rhizosphere N dynamics. Furthermore, there remains the daunting question of whether or not such re-tooled food crops can be developed and agronomically implemented in time to meet the swiftly approaching 2050 target date.

### Redesign farming systems

Farms are ecosystems, complex webs of biota interacting with one another and their habitat in countless interwoven flows of energy and matter. Because of the myriad of entanglements, there are few purely win-win options in managing ecosystems; usually, one must select among various tradeoffs (Meyfroidt et al., 2022). The expectation for delivery of innovative solutions and even the translation of assumed “existing innovations” to commercial settings inevitably leads to superficial technofixes with unanticipated side effects. Progress toward net-zero will require an understanding of all potential tradeoffs and synergies arising from prospective practices. This effort will almost certainly involve comprehensive computer models, which may include dynamic simulation modeling, statistical modeling to distill meaning from large datasets, and deployment of machine learning and related artificial intelligence approaches. These digital approaches, coupled with physical experimentation in laboratories, fields, and barns, are required to better understand interactions among system components and expose inherent uncertainties. This systems perspective also cautions against promising panacea to net-zero; all proposed strategies must be assessed through an analysis that considers all strands of required inputs (Wilson et al., 2024). For example, purported GHG savings from practices such as vertical farming, lab-grown meat, the “100 mile diet,” plant-based meat, and the use of agricultural biomass to generate fuel may not always be as clear-cut when system analyses are used to evaluate the circularity of these approaches (Box 3)

Box 3. **Examples of Agricultural Strategies That Have Been Proposed for Climate Mitigation**

Numerous agricultural strategies have been advocated in climate mitigation (Figure 6). How much these practices contribute to net-zero goals depends on: 1. The degree to which they reduce GHG emissions; and 2. The extent to which they can be adopted given economic, ecological, and social constraints. We have plotted some examples of possible practices using our own notional values. Ideally, an effective practice would effectively reduce emissions (Y-axis), with widespread adoption (X-axis). Very few practices fall into this upper-right quadrant. Regardless of the mitigation strategy, each has its own advantages and disadvantages and needs to be assessed from the perspective of the inevitable tradeoffs inherent to each technology.

### Re-imagine farming economics

Economic constraints may represent the single biggest obstacle to widespread adoption of systems that impel progress toward net-zero farming and promoting circularity. With rare exceptions, adopting the fundamental upheavals needed to substantively reduce net emissions will incur heavy economic costs (Edelenbosch et al., 2024) and jeopardize the short-term profitability of farms (Bowness et al., 2024). A net-zero strategy that does not promote thriving rural economies may never be widely, nor willingly, adopted. What will be needed, therefore, is a revamped economic structure that applies value (and financial rewards) to outputs beyond saleable commodities; farmers and ranchers may need economic returns, for example, for promoting C storage, biodiversity, or other non-market bioservices arising from multi-functional landscapes in new farming systems (Santos et al., 2021). Ideally, such structures would also reflect wider ethical and social aims beyond the strictly biophysical (Haring et al., 2023).

## Documenting Progress Toward Net-Zero Farming and Circularity

Setting targets for climate mitigation is meaningless, counterproductive, and distracting, without also establishing reliable, credible means for documenting progress toward set targets. Measuring net emissions and removals of GHGs across the vast diversity of farmlands is fraught with difficulty, but there are some steps that can be taken to document progress.

### Amplify process-based research to elucidate mechanisms of GHG emissions and removals from a circularity perspective

Despite intensive research in recent decades, many of the processes, mostly microbial, that underlie GHG emissions and C storage remain obscure. Particularly troublesome are the emissions of N_2_O, which exhibit enormous variability over time and space (Nevison et al., 2023), and our inadequate knowledge of the fundamental processes of enteric CH_4_ emissions and soil C dynamics. A richer, deeper understanding of underlying processes, arising from continuing research, may unveil factors affecting emissions and removals, thereby enhancing our understanding of both N and C cycles. Moreover, such emergent fundamental understanding may lead to better methods and strategies to measure net emissions across the vast and variable agricultural landscapes. In the haste to implement policy-driven practices, it is sometimes tempting to overlook the importance of the process-based science that will divulge better strategies and their evaluation.

### Plan and implement nationwide networks of long-term measurements

In the past, measurements of net GHG emissions have often involved brief, intensive studies, constrained by short-term funding arrangements. These scattered, sporadic pursuits have typically used inconsistent methodologies, focusing on single GHGs or farming sectors at localized sites. What is needed is a well-conceived network of monitoring sites representative of dominant farming systems across a nation, with a commitment to long-term continuance. Such a network might include sites both on commercial farms and at research centers, and employ consistent or at least inter-calibrated methodologies across all sites. Of particular importance, especially for monitoring soil C sinks, would be the collection and archiving of soil samples for future re-analysis using ever-improving methods (Liebig et al., 2024). To ensure wide accessibility and long-term reliability, GHG and circularity data, along with pertinent affiliated metadata, would be housed in a central repository in consistent, pre-determined formats.

### Maintain long-term expertise and a platform for iterative improvement of models

**Figure 5.**
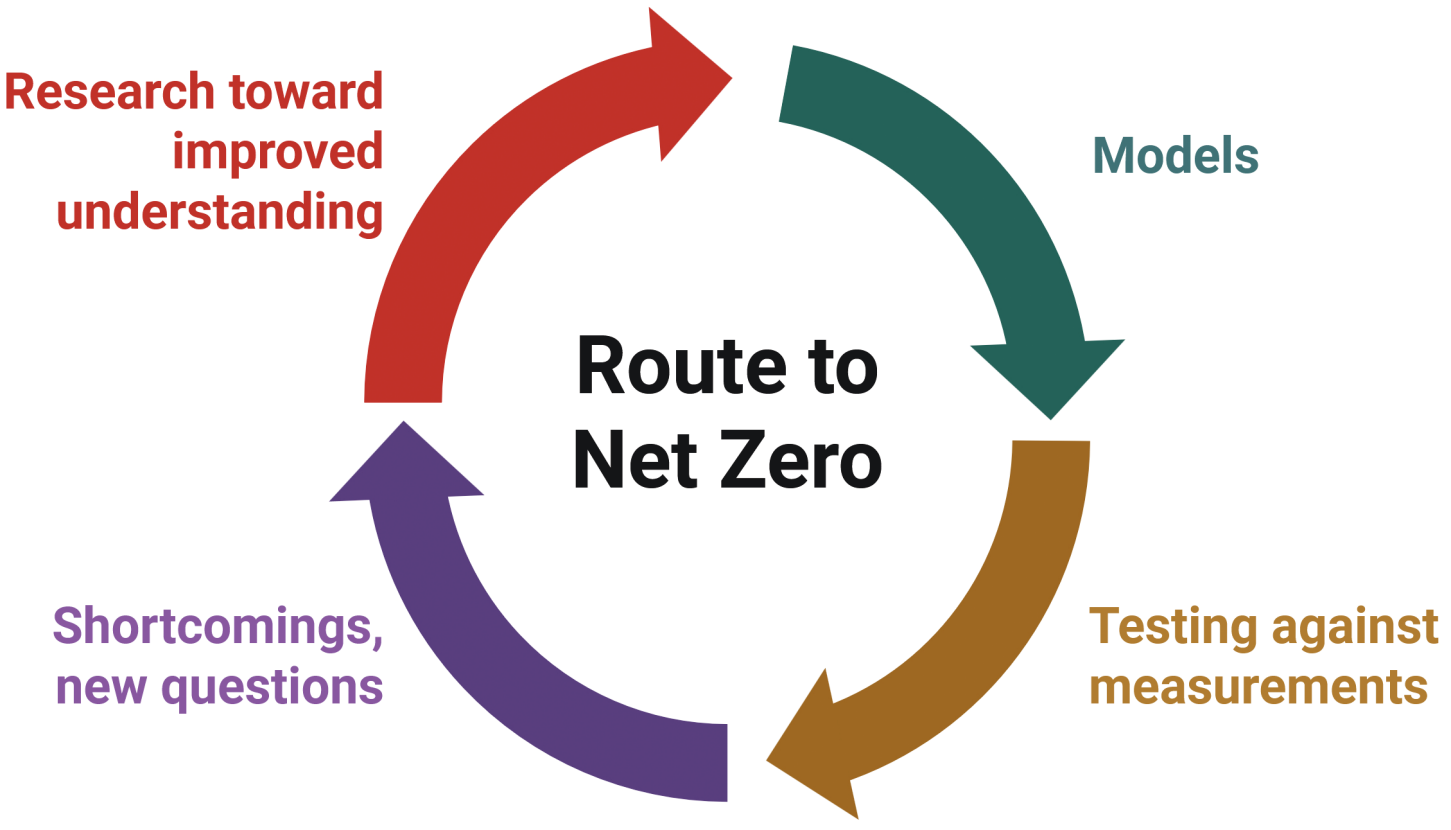
Iterative learning cycle in the development of improved models for estimating net GHG emissions. Understanding derived from research is used to create simulation models of varying complexity. Predictions from these models are tested against independent measurements, invariably disclosing shortcomings, leading to new questions that drive continuing research.

Given the variability and complexity of farming systems, computer models will invariably be needed to estimate net emissions of GHGs (Mattila et al., 2022) and circularity. To be credible, models must be continually verified and updated using data emerging from long-term measurements. Improved accuracy and precision of models, and their faithful rendering of underlying processes, may occur most efficiently through an iterative approach that involves a continual “learning cycle” that is increasingly aided by artificial intelligence (Figure 5).

**Figure 6.**
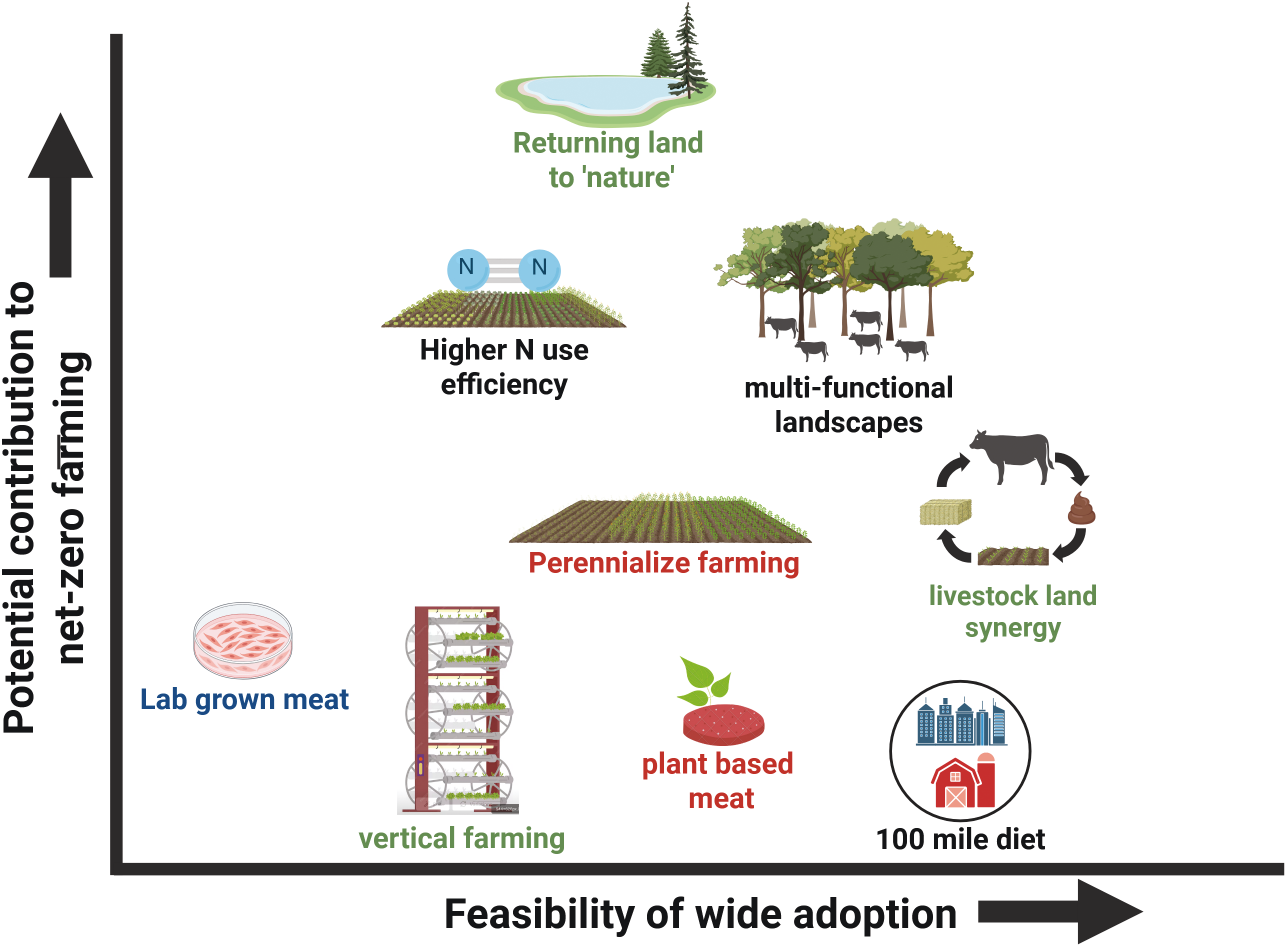
Examples of agricultural strategies that have been proposed for climate mitigation.

### Enhance communication among stakeholders

The contribution of enhanced circularity toward net-zero will depend on coordinated efforts from a wide array of participants: farmers, ranchers, policymakers, Indigenous communities, researchers, industry personnel, and consumers, among others (McGuire et al., 2023). Long-term investment in a campaign to document net GHG emissions, therefore, demands effective two-way communication about ongoing findings and prospects to ensure continuing reliability and accessibility of the results. Beyond merely delivering findings to satisfy policy targets, the monitoring strategy should also serve to mutually enlighten and encourage all participants in the ongoing process. A national strategy for documenting any progress toward net-zero farming will demand a long-term, substantive, and assured financial commitment. But without such a commitment, any target for reducing climate impact will be merely aspirational, a vague, notional commitment destined to fade away, yet another example of an “empty signifier,” whose motivating force diminishes as 2050 nears.

## Applicability of the Canadian Context to Global Agriculture

We used Canada as a case study, but with few exceptions, the principles and approaches discussed apply globally. Indeed, low- and middle-income countries, because of resource scarcity, may be more adept at implementing circularity practices than countries employing industrialized farming practices. Naturally, transformative change needs to be implemented with consideration for country-specific economic, environmental, and social constraints and opportunities. However, as farms are ultimately governed by biological and ecological principles, many transformative opportunities are applicable across agricultural systems. Providing incentives that encourage the adoption of transformative approaches tailored to country-specific farming practices represents an important step towards achieving global net-zero agricultural practices.

## Conclusions

We see no way of arriving at a durable net-zero target in agricultural systems by 2050 (and maintaining that status for ensuing decades), while also providing adequate food and other functions furnished by farmlands. Although this view may seem pessimistic, we remain, in fact, emphatically hopeful about the prospects of re-configuring farming to address climate change and other biospheric stresses. Enhancing circularity in agricultural systems will be a key step in this direction. Legitimate hope, always, is founded on an honest appraisal of where we are at now and how far we have yet to go in engaging the opportunities at hand (Figueres, 2024). Without realistically appraising the difficulty inherent in any GHG target, we are unlikely to muster the courage and creativity needed to substantively reduce emissions, let alone reach net-zero. Only sober acknowledgement of obstacles in the path to net-zero can lead us beyond wishful thinking toward hope. Like other environmental goals, the quest for climate-conscious farming confronts an apparent paradox, one that needs to be addressed with honesty and courage. Few questions, in our view, are more exciting, more hope-instilling—or more urgent. If the “net-zero farming by 2050” target offers the impetus to vigorously address such questions, then let us pursue it with fervent hope.

## About the Authors

**Tim McAllister** received an M. Sc. in Animal Biochemistry from the University of Alberta in 1987 and a Ph.D. in Microbiology and Nutrition from the University of Guelph in 1991. He has worked as a research scientist with Agriculture and Agri-Food Canada since 1996, where he now holds the position of principal research scientist in microbiology and beef cattle production. His team has addressed a variety of topic areas related to optimizing the role of livestock in a circular bioeconomy. These include assessing and mitigating greenhouse gas emissions from agricultural systems, optimizing manure management, utilizing food loss and waste as feed, promoting food safety, and recognizing the role of grassland ecosystems in sustaining biodiversity. His team has been the recipient of numerous societal awards for their contribution to beef cattle production in North America.

**Benjamin Ellert** earned a Ph. D. in soil science from the University of Saskatchewan in 1991. He has worked for Agriculture and Agri-Food Canada in Ottawa and Lethbridge, and since 1996 has been a Research Scientist in Biogeochemistry at the Lethbridge Research Centre in Southern Alberta. Currently, he serves as the Agroecosystem Science Team Lead. Ellert has extensive experience in carbon and nitrogen cycling in a diversity of agroecosystems and has a record of noteworthy contributions to the quantitative assessment of management-induced changes in soil carbon and nitrogen stocks, and of the land-atmosphere exchange of greenhouse gases. He has also contributed to the development and application of innovative isotopic techniques, at both natural abundance and artificially enriched levels, to tracing the circularity of C and N in agroecosystems.

**Henry Janzen** is a soil biochemist who has studied the flows of carbon and nutrients through farmlands, looking for ways to sustain their manifold functions. He has written extensively about the role of soils in mitigating climate change, participating in various international forums. Other topics he has pondered include soil nitrogen dynamics, whole-farm greenhouse gas modeling, and restorative cropping systems. More recently, he has contemplated how solar energy streams through our ecosystems, impelling their many and varied functions, ever-renewing their health. In all of these inquiries, Henry has sought a long-term narrative, reaching deep into the past by probing the soils’ memory, and peering ahead, trying to foresee how today’s choices shape the land for all its future inhabitants.

## References

[CIT0001] Allen, M.R., P. Friedlingstein, C.A.J. Girardin, S. Jenkins, Y. Malhi, E. Mitchell-Larson, G.P. Peters, and L. Rajamani. 2022. Net zero: science, origins, and implications. Annu. Rev. Environ. Resour. 47:849–887. doi: https://doi.org/10.1146/annurev-environ-112320-105050

[CIT0002] Battersby, S. 2024. “Net zero” may need a rethink to keep climate targets within reach. Proc. Natl. Acad. Sci. U.S.A. 121(18):e2407160121. doi: https://doi.org/10.1073/pnas.2407160121 38662547 PMC11066980

[CIT0003] Beans, C. 2022. Can countries expand agriculture without losing biodiversity? Weighing the options for feeding a growing world. BioScience 72(6):501–507. doi: https://doi.org/10.1093/biosci/biac030

[CIT0004] Beauchemin, K.A., E. Kebreab, M. Cain, and M.J. VandeHaar. 2024. The path to net-zero in dairy production: are pronounced decreases in enteric methane achievable? Annu. Rev. Anim. Biosci. 13(1):325–341. doi: https://doi.org/10.1146/annurev-animal-010324-113703 39546409

[CIT0005] Bowness, E., J. MacInnis, A.A. Desmarais, and S. Oke. 2024. Envisioning prairie agroecology: farmer visual constructions of place-based ecological agriculture in Canada. Elem. Sci. Anth. 12(1):00054. doi: https://doi.org/10.1525/elementa.2023.00054

[CIT0006] Cao, F., W. Li, Y. Jiang, X. Gan, C. Zhao, and J. Ma. 2024. Effects of grazing on grassland biomass and biodiversity: a global synthesis. Field Crops Res. 306:109204. doi: https://doi.org/10.1016/j.fcr.2023.109204

[CIT0007] Chapman, E.A., H.C. Thomsen, S. Tulloch, P.M.P. Correia, G. Luo, J. Najafi, L.R. DeHaan, T.E. Crews, L. Olsson, P.O. Lundquist, et al 2022. Perennials as future grain crops: opportunities and challenges. Front. Plant Sci. 13:898769. doi: https://doi.org/10.3389/fpls.2022.898769 35968139 PMC9372509

[CIT0008] Chiquier, S., A. Gurgel, J. Morris, Y.-H.H. Chen, and S. Paltsev. 2025. Integrated assessment of carbon dioxide removal portfolios: land, energy, and economic trade-offs for climate policy. Environ. Res. Lett. 20:024002. doi: https://doi.org/10.1088/1748-9326/ada4c0

[CIT0009] Christiansen, K.L., F. Hajdu, E.P. Mollaoglu, A. Andrews, W. Carton, and K. Fischer. 2023. “Our burgers eat carbon”: investigating the discourses of corporate net-zero commitments. Environ. Sci. Policy 142:79–88. doi: https://doi.org/10.1016/j.envsci.2023.01.015

[CIT0010] Desjardins, R.L., D.E. Worth, J.A. Dyer, X.P.C. Vergé, and B.G. McConkey. 2020. The carbon footprints of agricultural products in Canada. In: Muthu, S.S., editor. Carbon footprints: case studies from the building, household, and agricultural sectors. Singapore: Springer; p. 1–34, 2020. Print.

[CIT0011] Dooley, K. 2024. Net-Zero climate goals and the role of land-use. In: Fiorino, D.J., T.A. Eisenstadt, and M.K. Ahluwalia, editors. Elgar Encyclopedia of Climate Policy. Cheltenham (UK): Edward Elgar Publishing; pp. 302–305.

[CIT0012] Edelenbosch, O.Y., A.F. Hof, M. van den Berg, H.S. de Boer, H.-H. Chen, V. Daioglou, M.M. Dekker, J.C. Doelman, M.G.J. den Elzen, M. Harmsen, et al 2024. Reducing sectoral hard-to-abate emissions to limit reliance on carbon dioxide removal. Nat. Clim. Chang. 14(7):715–722. doi: https://doi.org/10.1038/s41558-024-02025-y

[CIT0013] Entz, M.H., and M. Van Die. 2024. Nurturing Canadian agronomy with nature: theory and practice. In: Yada, R.Y., R. Van Acker, M. Scanlon, and D. Gray, editors. Future Food Systems: Exploring Global Production, Processing, Distribution and Consumption. 1st ed. San Diego (CA): Academic Press; p. 3–16.

[CIT0014] Environment and Climate Change Canada. 2022. Exploring approaches for Canada’s transition to net-zero emissions [Long‑term low GHG emission development strategy]. United Nations Framework Convention on Climate Change (UNFCCC). [Accessed February 3, 2025]. https://unfccc.int/documents/620593.

[CIT0015] Environment and Climate Change Canada. 2024a. Canadian Environmental Sustainability Indicators: Greenhouse gas emissions. [Consulted on September 26, 2024; and Accessed February 3, 2025]. www.canada.ca/en/environment-climate-change/services/environmental-indicators/greenhouse-gas-emissions.html.

[CIT0016] Environment and Climate Change Canada (ECCC). 2024b. National inventory report 1990–2022: greenhouse gas sources and sinks in Canada. Part 1. Ottawa (ON): Environment and Climate Change Canada. [Accessed February 3, 2025]. https://publications.gc.ca/collections/collection_2024/eccc/En81-4-2022-1-eng.pdf.

[CIT0017] Food and Agriculture Organization of the United Nations (FAO). 2025. FAOSTAT Statistical Database. Rome (Italy): FAO. CC BY 4.0. [Accessed July 9, 2025]. https://www.fao.org/faostat/en/#data/RFN.

[CIT0018] Figueres, C. 2024. Why a mind-set of stubborn optimism about the climate crisis is needed, now more than ever. Bull. At. Sci. 80(1):38–40. doi: https://doi.org/10.1080/00963402.2023.2293576

[CIT0019] Franzluebbers, A.J., and J.R. Hendrickson. 2024. Should we consider integrated crop–livestock systems for ecosystem services, carbon sequestration, and agricultural resilience to climate change? Agron. J. 116(2):415–432. doi: https://doi.org/10.1002/agj2.21520

[CIT0020] Government of Canada. 2024. Net-zero emissions by 2050. Ottawa (ON): Government of Canada. [Accessed February 3, 2025]. https://www.canada.ca/en/services/environment/weather/climatechange/climate-plan/net-zero-emissions-2050.html.

[CIT0021] Green, J.F., and R. Salas Reyes. 2023. The history of net zero: can we move from concepts to practice? Environ. Polit. 32:901–915. doi: https://doi.org/10.1080/14693062.2023.2218334

[CIT0022] Guo, K., J. Yang, N. Yu, L. Luo, and E. Wang. 2023. Biological nitrogen fixation in cereal crops: progress, strategies, and perspectives. Plant Commun. 4(2):100499. doi: https://doi.org/10.1016/j.xplc.2022.100499 36447432 PMC10030364

[CIT0023] Haring, S., S. Pesci Schmulevich, G.M. Manser, and M.H. Cooper. 2023. Rethinking scientists’ ongoing participation in “feeding the world.” Front. Sustain. Food Syst. 7:1174704. doi: https://doi.org/10.3389/fsufs.2023.1174704

[CIT0024] IPCC. 2022. Annex I: Glossary [van Diemen, R., J.B.R. Matthews, V. Möller, J.S. Fuglestvedt, V. Masson-Delmotte, C. Méndez, A. Reisinger, S. Semenov (eds)]. In IPCC, 2022: Climate Change 2022: Mitigation of Climate Change. Contribution of Working Group III to the Sixth Assessment Report of the Intergovernmental Panel on Climate Change [P.R. Shukla, J. Skea, R. Slade, A. Al Khourdajie, R. van Diemen, D. McCollum, M. Pathak, S. Some, P. Vyas, R. Fradera, M. Belkacemi, A. Hasija, G. Lisboa, S. Luz, J. Malley, (eds.)]. Cambridge, UK and New York, NY, USA; Cambridge University Press; p. 803–829. doi: https://doi.org/10.1017/9781009157926.020

[CIT0057] Janzen H.H. 2011. Soil science on the Canadian prairies—Peering into the future from a century ago. Can. J. Soil Sci. 81(4):489–503. doi: https://doi.org/10.4141/S00-054

[CIT0025] Karimi, R., S.J. Pogue, R. Kröbel, K.A. Beauchemin, T. Schwinghamer, and H.H. Janzen. 2020. An updated nitrogen budget for Canadian agroecosystems. Agric. Ecosyst. Environ. 304:107046. doi: https://doi.org/10.1016/j.agee.2020.107046.

[CIT0026] Khalil, M.I., B.A. Osborne, and A. Wingler. 2024. Towards net zero emissions without compromising agricultural sustainability: What is achievable? Nutr. Cycl. Agroecosyst. 128(3):283–291. doi: https://doi.org/10.1007/s10705-024-10364-7

[CIT0027] Kremen, C., and A.M. Merenlender. 2018. Landscapes that work for biodiversity and people. Science 362(6412):eaau6020. doi: https://doi.org/10.1126/science.aau6020 30337381

[CIT0028] Kuzyakov, Y., and G. Domanski. 2000. Carbon input by plants into the soil. Z. Pflanzenernähr. Bodenk 163:421–431. doi: https://doi.org/10.1002/1522-2624(200008)163:4<421::AID-JPLN421>3.0.CO;2-R

[CIT0029] Li, Y., P. He, Y. Shan, Y. Li, Y. Hang, S. Shao, F. Ruzzenenti, and K. Hubacek. 2024. Reducing climate change impacts from the global food system through diet shifts. Nat. Clim. Change 14(9):943–953. doi: https://doi.org/10.1038/s41558-024-02084-1

[CIT0030] Liebig, M.A., F.J. Calderon, A.K. Clemensen, L. Durso, J.L. Duttenhefner, J.O. Eberly, J.J. Halvorson, V.L. Jin, K. Mankin, A.J. Margenot, et al 2024. Long-term soil change in the US Great Plains: an evaluation of the Haas Soil Archive. Agrosyst. Geosci. Environ. 7:e20502. doi: https://doi.org/10.1002/agg2.20502

[CIT0031] Liu, T., T. Huffman, S. Kulshreshtha, B. McConkey, Y. Du, M. Green, J. Liu, J. Shang, and X. Geng. 2017. Bioenergy production on marginal land in Canada: Potential, economic feasibility, and greenhouse gas emissions impacts. Appl. Energy 205:477–485. doi: https://doi.org/10.1016/j.apenergy.2017.07.126

[CIT0032] Mattila, T.J., E. Hagelberg, S. Söderlund, and J. Joona. 2022. How farmers approach soil carbon sequestration? Lessons learned from 105 carbon-farming plans. Soil Tillage Res. 215:105204. doi: https://doi.org/10.1016/j.still.2021.105204

[CIT0033] McGuire, R., S.A. Huws, C.H. Foyer, P. Forster, M. Welham, L. Spadavecchia, D. Curry, and N.D. Scollan. 2023. Agrifood and net zero. Trends Plant Sci. 28(5):495–497. doi: https://doi.org/10.1016/j.tplants.2023.02.011 36935267

[CIT0034] Meyfroidt, P., A. De Bremond, C.M. Ryan, E. Archer, R. Aspinall, A. Chhabra, and E.K. Zu Ermgassen. 2022. Ten facts about land systems for sustainability. Proc. Natl. Acad. Sci. U.S.A. 119(7):e2109217118. doi: https://doi.org/10.1073/pnas.2109217118 35131937 PMC8851509

[CIT0035] Monbiot, G. 2022. Regenesis: feeding the world without devouring the planet. New York: Penguin Books.

[CIT0036] Nevison, C., X. Lan, D. Worthy, and H. Tian. 2023. Top-down constraints on N2O emissions from Canada. Atmos. Environ. 313:120075. doi: https://doi.org/10.1016/j.atmosenv.2023.120075

[CIT0037] Nyberg, M., T.A. Black, R. Ketler, S.-C. Lee, M. Johnson, M. Merkens, J.A. Trofymow, and M. Ladd. 2022. Impacts of active versus passive re-wetting on the carbon balance of a previously drained bog. J. Geophys. Res. Biogeosciences 127:e2022JG006881. doi: https://doi.org/10.1029/2022JG006881

[CIT0038] Odum, E.P. 1969. The strategy of ecosystem development: an understanding of ecological succession provides a basis for resolving man’s conflict with nature. Science 164(3877):262–270. doi: https://doi.org/10.1126/science.164.3877.262 5776636

[CIT0039] Ogle, S.M., R.T. Conant, B. Fischer, B.K. Haya, D.T. Manning, B.A. McCarl, and T.J. Zelikova. 2023. Policy challenges to enhance soil carbon sinks: the dirty part of making contributions to the Paris agreement by the United States. Carbon Manag. 14(1):2268071. doi: https://doi.org/10.1080/17583004.2023.2268071

[CIT0040] Ominski, K., K. Gunte, K. Wittenberg, G. Legesse, G. Mengistu, and T. McAllister. 2021. The role of livestock in sustainable food production systems in Canada. Can. J. Anim. Sci. 101(4):591–601. doi: https://doi.org/10.1139/cjas-2021-0005

[CIT0042] Pankievicz, V.C., T.B. Irving, L.G. Maia, and J.M. Ané. 2019. Are we there yet? The long walk towards the development of efficient symbiotic associations between nitrogen-fixing bacteria and non-leguminous crops. BMC Biol. 17(1):99. doi: https://doi.org/10.1186/s12915-019-0710-0 31796086 PMC6889567

[CIT0043] Petit, S., and D.A. Landis. 2023. Landscape-scale management for biodiversity and ecosystem services. Agric. Ecosyst. Environ. 347:108370. doi: https://doi.org/10.1016/j.agee.2023.108370

[CIT0044] Prairie, A.M., A.E. King, and M.F. Cotrufo. 2023. Restoring particulate and mineral-associated organic carbon through regenerative agriculture. Proc. Natl. Acad. Sci. U. S. A. 120(21):e2217481120. doi: https://doi.org/10.1073/pnas.2217481120 37186829 PMC10214150

[CIT0045] Ripple, W.J., C. Wolf, J.W. Gregg, J. Rockström, M.E. Mann, N. Oreskes, T.M. Lenton, S. Rahmstorf, T.M. Newsome, C. Xu, et al 2024. The 2024 state of the climate report: Perilous times on planet Earth. BioScience 74(12):812–824. doi: https://doi.org/10.1093/biosci/biae087

[CIT0046] Rogelj, J. 2023. Net zero targets in science and policy. Environ. Res. Lett. 18(2):021003. doi: https://doi.org/10.1088/1748-9326/acb4ae

[CIT0047] Santos, J.L., F. Moreira, P.F. Ribeiro, M.J. Canadas, A. Novais, and A. Lomba. 2021. A farming systems approach to linking agricultural policies with biodiversity and ecosystem services. Front. Ecol. Environ. 19(3):168–175. doi: https://doi.org/10.1002/fee.2292

[CIT0051] Soto-Gomez, D. 2024. Integration of crops, livestock, and solar panels: a review of agrivoltaics systems. Agronomy 14(8): 1824. doi: https://doi.org/10.3390/agronomy14081824

[CIT0048] Sprunger, C.D., P. Singh, and T. Martin. 2024. Chapter 9 - Integrating perennials into agroecosystems for enhanced soil biodiversity and long-term sustainability. In: Singh K., M. C. Ribeiro, and Ö. Calicioglu, editors. Biodivers. Bioeconomy. Oxford (UK): Elsevier; p. 199–216. doi:doi: https://doi.org/10.1016/B978-0-323-95482-2.00009-2

[CIT0049] Statistics Canada. 2024. Land use, Census of Agriculture, 2021 (Table 32‑10‑0249‑01). Statistics Canada Catalogue number. [Accessed July 9, 2025]. https://www150.statcan.gc.ca/t1/tbl1/en/tv.action?pid=3210024901.

[CIT0050] Steffen, W., K. Richardson, J. Rockström, S.E. Cornell, I. Fetzer, E.M. Bennett, and S. Sörlin. 2015. Planetary boundaries: guiding human development on a changing planet. Science 347(6223):1259855. doi: https://doi.org/10.1126/science.1259855 25592418

[CIT0052] Sutton, W. R., A. Lotsch, and A. Prasann. 2024. Recipe for a Livable Planet: Achieving Net Zero Emissions in the Agrifood System. Agriculture and Food Series. © Washington, DC: World Bank. http://hdl.handle.net/10986/41468.

[CIT0053] Taylor, S., P.J. Gilbert, D.A. Cooke, M.E. Deary, and M.J. Jeffries. 2019. High carbon burial rates by small ponds in the landscape. Front. Ecol. Environ. 17(1):25–31. doi: https://doi.org/10.1002/fee.1988

[CIT0041] Van Oort, B., A.S. Daloz, R. Andrew, F.M. Farstad, M. Guillen-Royo, E.A.T. Hermansen, N.B. Holmelin, S. Kallbekken, A. Orlov, J. Sillmann, et al 2024. Ruminating on sustainable food systems in a net-zero world. Nat. Sustain. 7(10):1225–1234. doi: https://doi.org/10.1038/s41893-024-01404-9

[CIT0054] Whitmee, S., R. Green, K. Belesova, S. Hassan, S. Cuevas, P. Murage, R. Picetti, R. Clercq-Roques, K. Murray, J. Falconer, et al 2024. Pathways to a healthy net-zero future: report of the Lancet Pathfinder Commission. Lancet 403(10421):67–110. doi: https://doi.org/10.1016/S0140-6736(23)02466-2 37995741

[CIT0055] Wilson, K.R., M.K. Hendrickson, and R.L. Myers. 2024. A buzzword, a “win-win”, or a signal towards the future of agriculture? A critical analysis of regenerative agriculture. Agric. Hum. Values 42(1):257–269. doi: https://doi.org/10.1007/s10460-024-10603-1

[CIT0056] Zickfeld, K., A.J. MacIsaac, J.G. Canadell, S. Fuss, R.B. Jackson, C.D. Jones, A. Lohila, H.D. Matthews, G.P. Peters, J. Rogelj, et al 2023. Net-zero approaches must consider Earth system impacts to achieve climate goals. Nat. Clim. Change 13(12):1298–1305. doi: https://doi.org/10.1038/s41558-023-01862-7

